# Machine Learning Enabled P300 Classifier for Autism Spectrum Disorder Using Adaptive Signal Decomposition

**DOI:** 10.3390/brainsci13020315

**Published:** 2023-02-13

**Authors:** Santhosh Peketi, Sanjay B. Dhok

**Affiliations:** Center for VLSI and Nanotechnology, Visvesvaraya National Institute of Technology, Nagpur 440010, India

**Keywords:** autism spectrum disorder (ASD), brain–computer interface (BCI), P300 electroencephalogram (EEG) signal, machine learning (ML), variational mode decomposition (VMD)

## Abstract

Joint attention skills deficiency in Autism spectrum disorder (ASD) hinders individuals from communicating effectively. The P300 Electroencephalogram (EEG) signal-based brain–computer interface (BCI) helps these individuals in neurorehabilitation training to overcome this deficiency. The detection of the P300 signal is more challenging in ASD as it is noisy, has less amplitude, and has a higher latency than in other individuals. This paper presents a novel application of the variational mode decomposition (VMD) technique in a BCI system involving ASD subjects for P300 signal identification. The EEG signal is decomposed into five modes using VMD. Thirty linear and non-linear time and frequency domain features are extracted for each mode. Synthetic minority oversampling technique data augmentation is performed to overcome the class imbalance problem in the chosen dataset. Then, a comparative analysis of three popular machine learning classifiers is performed for this application. VMD’s fifth mode with a support vector machine (fine Gaussian kernel) classifier gave the best performance parameters, namely accuracy, F1-score, and the area under the curve, as 91.12%, 91.18%, and 96.6%, respectively. These results are better when compared to other state-of-the-art methods.

## 1. Introduction

Autism spectrum disorder (ASD) is a group of persistent and pervasive neurodevelopmental conditions. It is characterized by enduring difficulties with social engagement, communication and a constrained, recurrent behavior, interest, or activity pattern [1]. As per the World Health Organization (WHO), one among 100 children has ASD worldwide [2]. According to the United States Centers for Disease Control and Prevention (CDC) Autism and Developmental Disabilities Monitoring (ADDM) Network, 1 in 54 children have been diagnosed with ASD [3]. People with ASD are known to have severe joint attention (JA) deficiency [4]. It is crucial for the growth of their social and linguistic skills [5]. JA is an important social communication skill that starts developing in the early stages of life. It is characterized by the nonverbal coordination of two people’s attention toward a third thing or event. Simply put, joint attention refers to a variety of behaviors involving another person and is related to attention (dyadic and triadic).

A review by Friedrich et al. [6] suggested that electroencephalogram (EEG)-based neurofeedback training is viable as a personalized therapeutic approach to ASD. Keeping this aspect in mind, Amaral and Simoes et al. [7] conducted a clinical trial to assess the feasibility of their brain–computer interface (BCI) system based on a visual P300 signal for the enhancement of JA skills in ASD subjects.

A BCI is a closed-loop system that takes brain EEG signals, analyzes them, and translates them into commands for the operation of physical or virtual systems that carry out intended actions without the requirement of any physical activity from the user [8]. BCI systems use EEG signals, which are the electrical potentials generated by the brain activity and are collected non-invasively by the electrodes placed on the scalp. The temporal resolution of the EEG signal is high, and its acquisition devices are cost-effective compared to other brain imaging techniques and are portable. These advantages encourage researchers to develop various applications of BCI in real life, such as wheelchair control [9], gaming control [10], neurorehabilitation training [11], and a spelling system [12], only to name a few. Recent and interesting applications such as controlling robots using EEG signal integration [13], using common spatial patterns on EEG for robotic arm control [14], the application of domain adaptation techniques such as Riemannian manifold on the EEG signal to decrease the calibration time in motor imagery BCI [15] were realized based on EEG signal analysis in combination with BCI technology.

Machine learning (ML) classifiers were found to achieve state-of-the-art performance in classifying and dealing with non-linear EEG signal data. They could arrive at complex decision boundaries to separate different classes of data without needing any human intervention. ML classifiers can quickly identify the trends and patterns of the data. In recent times, many innovative EEG applications in ML-based disease and mental load prediction have been realized. An ML-based system was designed for a real-time health monitoring system to classify healthy individuals and patients with the prognostics of an ischemic stroke using an ambulatory EEG system in [16]. An ML-based classifier was used in [17] to identify individuals with ischemic stroke from a set of healthy individuals by evaluating the EEG biomarkers. An ML-based system was realized for the classification of two opposing cognitive groups composed of those who regularly engage in rumination as a negative emotion regulation technique and those who engage in it less frequently [18]. Berke Kılıç et al. [19] resorted to ML techniques to classify emotional states using EEG signals. Xiang Liu et al. [20] used a deep forest ML classifier and variational mode decomposition for the detection of epileptic seizures from EEG signals. All these applications indicate the efficacy of ML classifiers in EEG applications.

### 1.1. Motivation

Most of the BCI can be based on event-related potential (ERP), sensory-motor rhythms (SMR), slow cortical potentials (SCP), or study state visually evoked potentials (SSVEP). BCI, based on the P300 signal, is one of the most renowned systems used in many applications. The detection of P300 is vital in building up a practically useful BCI system. P300 is a positive deflection in the EEG of central and parietal electrodes around 300 ms after the onset of a visual stimulus [21]. P300 is an ERP that is mostly elicited in an odd-ball paradigm [22] when a person is paying attention to a rare stimulus, among several others. P300 signals are weak signals and are usually embedded with many interferences such as background noise, muscular movements, power line noise, and corneoretinal standing potentials; as a result, the signal-to-noise (SNR) is poor. It makes the detection of the P300 signal difficult. Ensemble averaging [23] is generally performed to improve the SNR. Nevertheless, by doing so, within-subject variance becomes lost [24]. Also, P300 component detection by the visual inspection of the grand-average waveforms can be highly unreliable as it is usually erroneous and sensitive to experimental bias [24].

**Figure 1:**
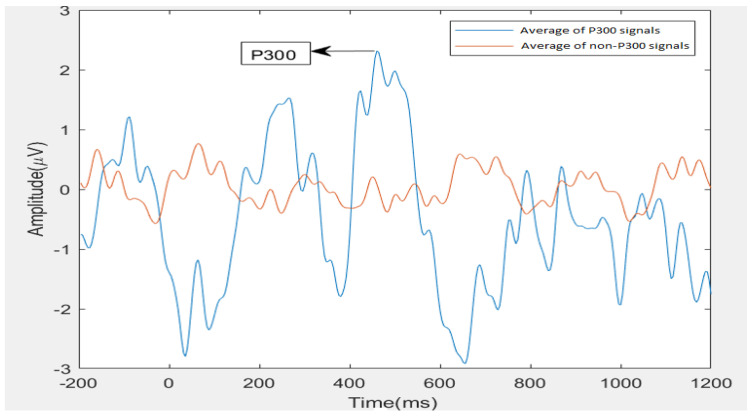
ASD P300 and non-P300 grand averaged signal from the Pz channel.

A recent study by Jitender Sorout et al. [25] to access the normative data of the P300 amplitude and latency on hundred healthy individuals in the age group of (18–25) years with a mean age of 19.81±1.80 years revealed that the mean P300 latency and amplitude were 281.38±33.39 ms and 4.53±1.67 μV, respectively. Figure 1 shows the grand average of P300 and non-P300 signal amplitudes from the Pz channel for an individual with ASD in the dataset considered in this research work. Here from −200 ms to 0 ms is the pre-stimulus interval, and the stimulus is onset at 0 ms.

By visual inspection, it can be observed that the P300 wave’s amplitude is <2.5 μV, and latency is >400 ms. It shows that the P300 amplitude is less and latency is more in ASD when compared to healthy individuals. This atypicality of the P300 response is due to cognitive deficiencies in ASD individuals. By this, it is evident that it is challenging to detect the P300 signal in ASD individuals when considered at the single trial level. As EEG is inherently susceptible to various unavoidable artifacts, efficient signal processing methods accompanied by robust classifiers are required to detect P300 signals in ASD individuals. A meta-analysis by Tingkai Cui et al. [26] proved a decrease in the P300 amplitude of ASD subjects compared to typically developing (TD). An analysis by Tanu et al. [27] also showed latency in the occurrence of P300 signals and a decrease in the amplitude of the P300 signal. It shows that recognition of P300 in ASD subjects is complicated.

In the previously proposed methods for the detection of P300 in ASD subjects, Zhao et al. [28] employed linear discriminant analysis (LDA), support vector machine (SVM), and convolutional neural network (CNN) with a novel and personalized filter design and obtained an accuracy of 67.2%. Adama et al. [29] used time domain features and the Pearson correlation coefficient to obtain an accuracy of 70%. Bipra Chatterjee et al. [30] leveraged temporal features and used three classifiers, Bayes LDA, random under-sampling boosting (RUSBOOST), and CNN, out of which BLDA outperformed others, giving an accuracy of 76.3%. Miladinovic et al. [31] applied logistic regression based on variational Bayesian inference (VB-ARD) and obtained an accuracy of 80.3%. Bittencourt-Villalpando et al. [32] trained linear LDA using augmented data obtained by pseudo-random averaging and achieved an accuracy of 81.2%. Lucia de Arancibia et al. [33] extracted time domain and CWT features from the data and explored LDA, linear SVM (LSVM), and radial SVM (RSVM) classifiers. Among these, LDA gave better accuracy of 82%. Santamaría-Vázquez et al. [34] utilized CNN- bidirectional long short-term memory (CNN-BLSTM) and achieved an accuracy of 84.3%. Borra et al. [35] used CNN based on the EEG-NET architecture [36] proposed by Lawhern et al. and obtained an accuracy of 92.3%. None of the previous approaches in the literature explored the application of adaptive signal decomposition techniques for P300 classification in ASD subjects. Hence, the present study explored the adaptive signal decomposition method for P300 classification in a BCI for ASD.

Due to the non-stationary nature of EEG data, the analysis of these signals is difficult. Fourier transform (FT) gives information on the frequency content of the signal, but it is not able to convey information about where in time, the actual frequency components appear. Moreover, FT is unsuitable for non-stationary signal analyses such as EEG. Short-time Fourier transform (STFT) techniques rely on selecting the proper window function. The shorter window size in STFT results in an excellent time resolution but poor frequency resolution. A larger window size would produce a better frequency resolution but, in contrast, a worse time resolution. Arriving at a consensus between the time and frequency resolution is difficult in STFT, and it’s a primary disadvantage. Wavelet transform (WT) has a good time-frequency resolution but is complex and computationally expensive. In order to overcome the disadvantages of the above-said techniques, many researchers have resorted to adaptive signal decomposition techniques.

Adaptive signal decomposition techniques are data-driven, and there is no necessity to know the nature of the basis function to match the signal characteristic of a priory. They represent a given signal into its constituent modes [37] and a residue representing an arbitrary signal’s oscillatory property. Some of the popular decomposition techniques existing in the literature are empirical mode decomposition (EMD), empirical wavelet transform (EWT), and variational mode decomposition (VMD).

The EMD [37] method decomposes the signal into principal modes but entirely depends on the stopping criterion imposed, extremal point finding methods, and extremal points interpolation into carrier envelopes. EMD also lacks a strong mathematical theory in its design. EWT [38] builds an adaptive wavelet basis for the signal’s adaptive sub-band decomposition, but the frequency band construction seems slightly rigid. Dragomiretskiy et al. proposed a more popular decomposition technique named VMD [39]. VMD overcomes the shortcomings of EMD’s lack of mathematical foundation, sensitivity to noise, and EWT’s strict filter bank boundaries. Recently, Ashwin Kamble et al. [40] used VMD for BCI-based silent communication and arrived at a good classification accuracy compared to other algorithms such as EMD, EWT, and variational nonlinear chirp mode decomposition(VNCMD). Smith K. Khare et al. [41] used VMD for emotion recognition.

### 1.2. Novelty and Major Contributions

This work proposes a novel VMD-based approach for classifying P300 signals in ASD subjects using ML classifiers. To the best of our knowledge, none of the previous researchers have explored the VMD adaptive signal decomposition method for P300 EEG signal classification in a BCI system involving individuals with ASD using ML classifiers. The proposed method was tested on probably the only publicly available benchmark BCIAUT-P300 multisession dataset collected from individuals with ASD. This suggested approach might be utilized to create useful BCI to assist people with ASD.

The major contributions of this paper are:

- It is the first study to evaluate the usefulness of the VMD method for classifying P300 and non-P300 signals in ASD subjects.
- The performance of three popular ML algorithms belonging to three different categories is evaluated, and a better-performing one is recommended for the proposed method.
- Mode-wise comparison is performed for the VMD-ASD application to select the best mode with optimal classification performance.
- Improved classification performance is achieved compared to existing state-of-the-art techniques reported in the literature.

## 2. Materials and Methods

### 2.1. BCIAUT-P300 Dataset for ASD

The BCIAUT-P300 dataset [42] considered in this work is probably the only publicly available P300-based benchmark BCI dataset for ASD individuals. Given below are the details of EEG data acquisition.

#### 2.1.1. Participants of the Experiment

The dataset was collected from 15 subjects with ASD. The participants were in the age group of sixteen to thirty-eight years, and their average age was twenty-two years and two months.

#### 2.1.2. EEG Data Acquisition Process

**Figure 2:**
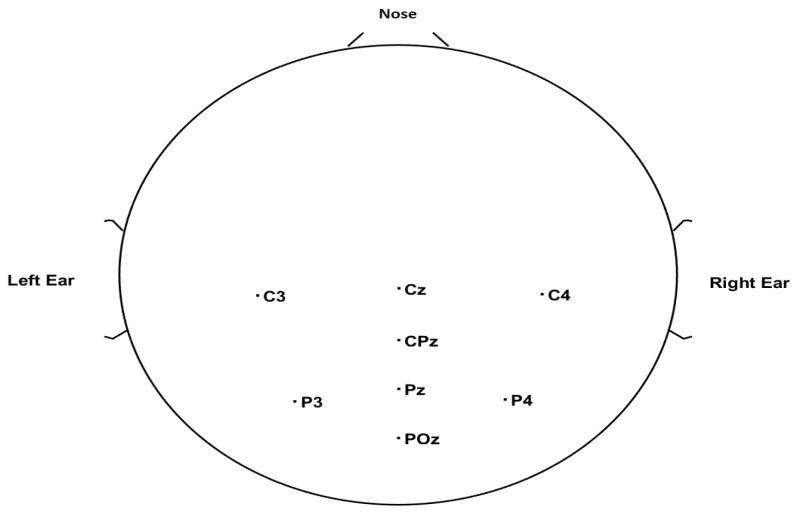
EEG channel locations considered for the dataset experiment.

The EEG data were recorded using g.Nautilus (gTEC, Austria) wireless equipment from eight central and parietal electrodes C3, Cz, C4, CPz, P3, Pz, P4, and POz. The ground electrode was placed at the AFz electrode position and the reference electrode at the right ear. The top view of the EEG electrode layout in accordance with the international 10–20 system of the electrode placement is shown in Figure 2. The EEG sampling frequency is 250 Hz.

#### 2.1.3. Experimental Design and Stimulus Parameters

**Figure 3:**
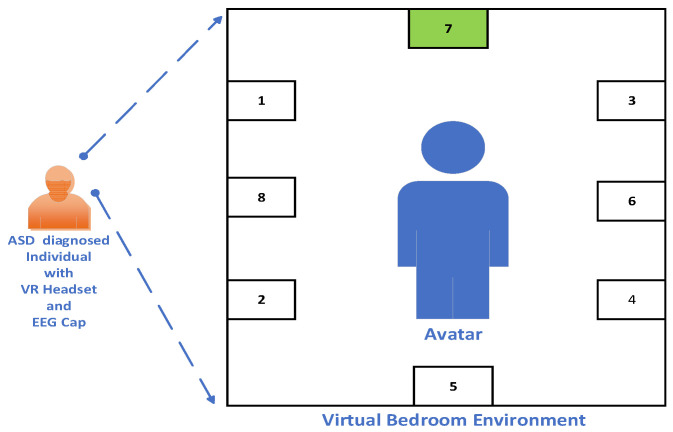
Illustration of the dataset VR environment.

The ASD individuals involved in the experiment underwent seven identical training sessions. The initial four sessions were conducted weekly, and the last three sessions were conducted monthly. The ASD subjects were equipped with a VR headset and an EEG Cap to record their EEG signals [42]. The VR environment is a virtual bedroom scene with a virtual character (an Avatar) inside it. A sample illustration of the dataset VR environment is shown in Figure 3. The bedroom VR environment consists of eight objects which are used as stimuli for the experiment to train and carry out the proposed task. The objects were labeled as 1 (books), 2 (a radio), 3 (a printer), 4 (laptop), 5 (a ball), 6 (cardboard), 7 (wooden plane), and 8 (a picture) [42]. During the experiment, the ASD individuals had to concentrate on the head movement (head cue) of the Avatar, which was looking at one of the eight objects (target object) in the VR environment (joint attention task). The EEG data are collected from them in two different phases, called calibration and online phases. A detailed explanation of these phases is given as follows:

##### Calibration Phase

**Figure 4:**
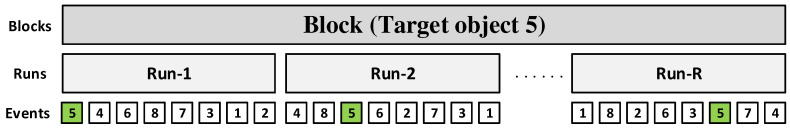
Block, run and event structure of the dataset experiment.

The EEG data from the dataset experiment is collected in the form of blocks runs, and events [42]. Each session data in this phase consists of 20 blocks, each for 10 different runs of the experimental condition. Each run comprises eight different objects; each one flashes with the green light in a randomized manner. Each object’s highlight (flash) appears with a 200 ms inter-stimulus interval. Each flash lasted 100 ms. The pictorial illustration of blocks, runs, and events is shown in Figure 4. During this phase, the participants were explicitly instructed to count the occurrences of one of the already specified objects that would flash during each block. The target object was the one that was clearly mentioned to the participant during this step. The participants were asked to confirm the object after each run (behavioral control). The data collected during this phase are called training data.

##### P300 Occurance

A pseudorandom number generator was used to choose the target item in each block (i.e., 1 to 8) [42]. The experimenter explained the target object to the participants, who were told to track how many times it flashed. This approach induces a rare event with a 1/8 target event probability, resulting in a P300 brain response.

##### Online Phase

Each session data in this phase consisted of 50 blocks, and each block consisted of a number of runs that varied from 3 to 10 and were selected based on the calibration phase. Each block run has a similar structure to the calibration phase. In the online phase, the target object is not informed to the ASD individual. The participant needs to follow the head movement of the Avatar instantaneously and pay attention to the target object in the VR environment, and also count the number of times it has flashed. The data collected during this phase are called testing data. The detailed explanation of the structure of the training and testing data, along with their related events, targets, and label information, is given in detail in [42].

#### 2.1.4. Data Pre-Processing

The raw EEG signals in the BCIAUT-P300 dataset are properly pre-processed by different signal processing techniques to remove artifacts [42].

##### Filtering the EEG Signals

The EEG signals are notch filtered at 50 Hz to eliminate the powerline noise. As the EEG was acquired only from C3, Cz, C4, CPz, P3, Pz, P4, and POz electrode positions, which are located at the central and parietal regions of the brain, there was no effect observed by the EOG artifacts, which arose from the eye movements and blinks. ECG artifacts are, in general, pulse artifacts that may interfere with EEG signals in the frequency range of around (0–1.2 Hz). Hence, the EEG signals are bandpass filtered between 2 and 30 Hz. By doing so, the ECG artifacts are eliminated, and the P300 EEG frequencies, which usually occur in frequencies below 30 Hz, are retained.

##### EEG Signal Data Manipulation for Classification

In order to prepare the data for this research work, the baseline period data samples, which occur 200 ms before the onset of the stimulus, are removed from the EEG event data. Then, 250 samples are extracted from the time of stimulus initiation from 0 ms to 1000 ms later. Eight average signals are generated for each block by averaging together all of the EEG events that pertain to the same object inside that block. The EEG events corresponding to the training and testing data of all seven sessions were concatenated to obtain 8×250×3920 events/epochs. Here, 8 represents the number of channels, 250 represents the number of time samples, and 3920 represents the number of epochs. Each channel’s data were normalized by subtracting the mean of the respective channel data and then dividing the result obtained by the standard deviation of the respective channel data to obtain the final dataset. The epochs in the 3-dimensional space were vertically concatenated to obtain a 2-dimensional matrix of the size 31,360×250 so as to make the dataset compatible with VMD.

### 2.2. Proposed VMD-SVM Method

**Figure 5:**
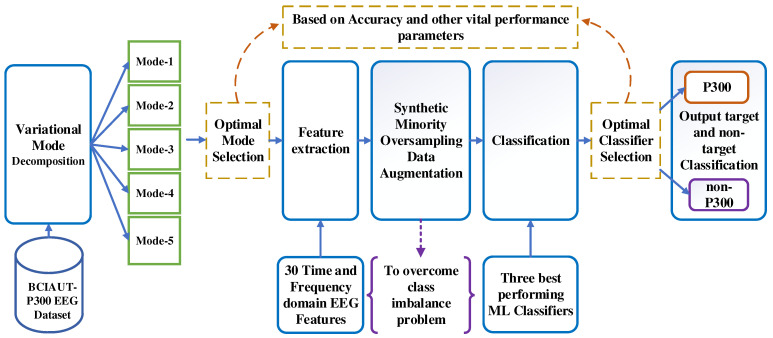
Various stages of the proposed VMD-SVM method.

Owing to the nonlinear and non-stationary nature of the EEG signals, fixed time and frequency-based analysis techniques might not extract the relevant information needed from the signal. Hence, this study considered VMD to analyze EEG signals as it is one of the most popular adaptive signal processing techniques. The outline of the proposed model is shown in Figure 5. The various stages of the proposed VMD-SVM method are explained below.

#### 2.2.1. VMD Decomposition

VMD is highly robust to noise and signal sampling. Hence, VMD was chosen for our application. VMD is an adaptive signal decomposition technique that is used to decompose a signal, say x(t), into a finite number of sub-signals called intrinsic mode functions (IMFs) [34] or simply modes which are amplitude-modulated-frequency-modulated (AM-FM) signals. They have specific sparsity properties when reproducing the input signal. The sparsity prior to each mode is chosen as the spectral domain bandwidth. The bandwidth of each mode is estimated in the following procedure: (i) the unilateral frequency spectrum is obtained by computing the corresponding analytic signal using the Hilbert transform for each mode. (ii) The shift in the frequency spectrum of each mode to the baseband is achieved by mixing with an exponential tuned to the approximated center frequency for each mode. (iii) At last, the bandwidth is estimated by calculating the squared $L^{2}$-norm of the gradient. The modes are obtained by solving the following constrained variational problem [39] below.

(1) $$\begin{array}{c} \min_{\left\{s_{m}\right\},\left\{\omega_{m}\right\}}\left\{\sum_{m}{∥\partial_{t}\left[\left(\delta \left(t\right)+\frac{j}{\pi t}\right)*s_{m}\left(t\right)\right]e^{-j\omega_{m}t}∥}_{2}^{2}\right\}\\ \;s.t.\;\;\sum_{m}s_{m}\left(t\right)=x\left(t\right) \end{array}$$

where $\partial_{t}$ represents the partial derivative, *j* is an imaginary number, δ(t) is an impulse function, $s_{m}\left(t\right)$, and $\omega_{m}$ indicate the $m^{th}$ decomposed mode and its respective center frequency in (1). To convert the above-constrained problem to an unconstrained, quadratic penalty term (α), Lagrange multiplier (λ) and augmented Lagrangian (L) are introduced [39]. The unconstrained optimization problem can then be denoted as:

(2) $$\begin{array}{cc} L & \left(\left\{s_{m}\right\},\left\{\omega_{m}\right\},\lambda \right):=\alpha \sum_{m}{∥\partial_{t}\left[\left(\delta \left(t\right)+\frac{j}{\pi t}\right)*s_{m}\left(t\right)\right]e^{-j\omega_{m}t}∥}_{2}^{2}\\ \; & +{∥x\left(t\right)-\sum_{m}s_{m}\left(t\right)∥}_{2}^{2}+\langle \lambda \left(t\right),x\left(t\right)-\sum_{m}s_{m}\left(t\right)\rangle \end{array}$$

By solving (2) using the sub-optimizations called the alternate direction of multipliers (ADMM) [39], the following equations for the mode update and center of frequency update equations are obtained [39].

(3) $${\hat{s}}_{m}^{n+1}\left(\omega \right)=\frac{\hat{x}\left(\omega \right)-\sum_{i<m}{\hat{s}}_{i}^{n+1}\left(\omega \right)-\sum_{i>m}{\hat{s}}_{i}^{n}\left(\omega \right)+\frac{{\hat{\lambda }}^{n}\left(\omega \right)}{2}}{1+2\alpha {\left(\omega -\omega_{m}^{n}\right)}^{2}}$$

(4) $$\omega_{m}^{n+1}=\frac{\int_{0}^{\infty }\omega {\left|{\hat{s}}_{m}^{n+1}\left(\omega \right)\right|}^{2}d\omega }{\int_{0}^{\infty }{\left|{\hat{s}}_{m}^{n+1}\left(\omega \right)\right|}^{2}d\omega }$$

The procedure that is followed for the VMD algorithm can thus be summarized as follows:

- Initialize the values of {${\hat{s}}_{m}^{1}$}, {$\omega_{m}^{1}$},{${\hat{\lambda }}^{1}$}, and keep n = 0;
- Update the ${\hat{s}}_{m}$ and $\omega_{m}$ as per (3) and (4);
- Update the dual ascent λ using:
  (5) $${\hat{\lambda }}^{n+1}\left(\omega \right)\leftarrow {\hat{\lambda }}^{n}\left(\omega \right)+\tau \left(\hat{x}\left(\omega \right)-\sum_{m}{\hat{s}}_{m}^{n+1}\left(\omega \right)\right)$$
- Iterate the steps (ii) and (iii) until convergence:
  (6) $$\sum_{m}{∥s_{m}^{n+1}-s_{m}^{n}∥}_{2}^{2}/{∥s_{m}^{n}∥}_{2}^{2}<\epsilon$$

**Figure 6:**
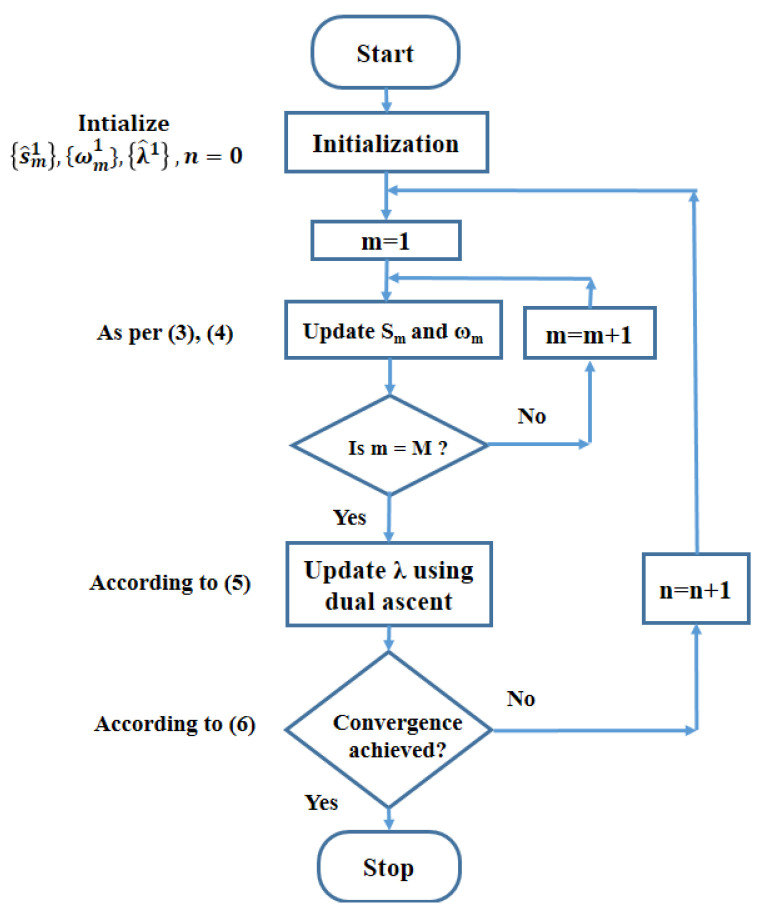
Flowchart of VMD algorithm.

The detailed working of the VMD algorithm is explained clearly in the form of a flowchart in Figure 6. Here M refers to the number of modes.

**Figure 7:**
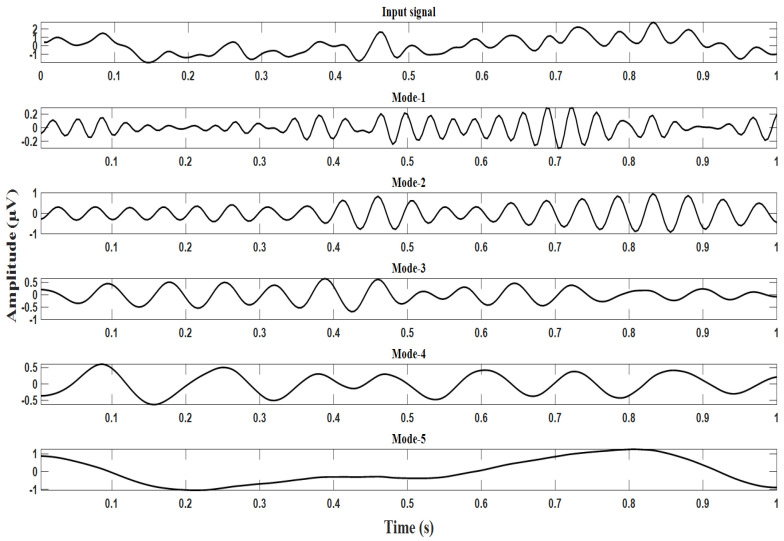
Decomposition of non-target EEG signals using VMD.

**Figure 8:**
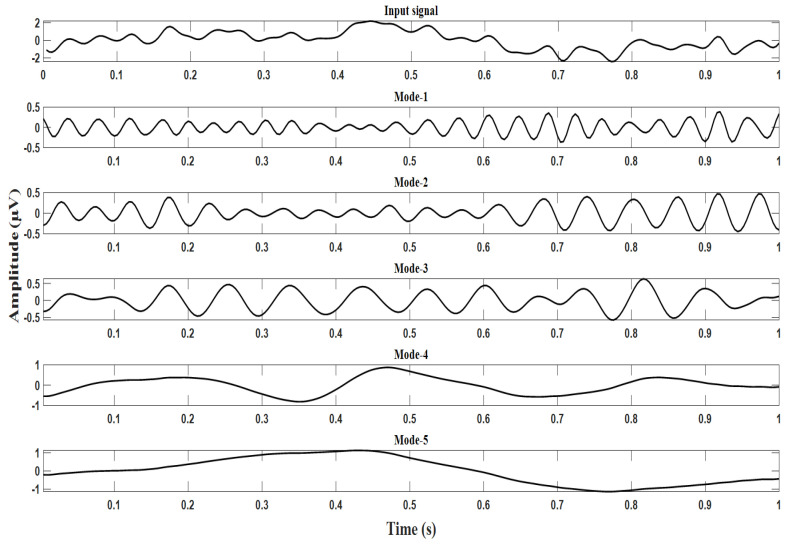
Decomposition of target EEG signals using VMD.

For our application, the VMD algorithm parameters’ relative tolerance ϵ=0.005, Lagrange multiplier update rate τ=0.01, α=1000, and M = 5 were chosen. As the EEG signals are generally classified in the range of frequencies delta (1–3 Hz), theta (4–7 Hz), alpha (8–12 Hz), beta (13–30 Hz), and gamma (30–100 Hz), VMD is similarly applied to the pre-processed final dataset matrix, and five IMFs on the above ranges were extracted for each channel data. The resulting IMFs are designated Mode-1, Mode-2, Mode-3, Mode-4, and Mode-5, respectively. The specimen input waveforms for the target’s P300 and non-targets non-P300 signals, along with their modes, are shown in Figure 7 and Figure 8. It can be observed from the Mode-5 signal of Figure 8 that the P300 signal is more pronounced, and its peak occurs almost after a latency from 300 ms to 500 ms and the stimulus onset. On the other hand, the P300 signal is not found in the range from 300 ms to 500 ms and in the Mode-5 signal of Figure 7.

#### 2.2.2. Feature Extraction

For each mode, thirty linear and non-linear time and frequency domain features such as the mean, median, standard deviation, kurtosis, skewness, first difference, normalized first difference, second difference, normalized second difference, Hjorth Activity, Hjorth Mobility, Hjorth complexity, entropy, Log Energy Entropy, log root sum of sequential variation, maximum, minimum, mean curve length, mean energy, mean Teager energy, Shannon entropy, Renyi entropy, Tsallis entropy, skewness, band power alpha, band power beta, band power gamma, band power theta, band power delta, and the ratio of band power alpha beta [43,44,45] were extracted and used to train machine learning (ML) classifiers. These features were found to give maximum statistical variance when tested using the Kruskal–Wallis (KW) test and hence were selected. The KW test is a non-parametric method to test whether the samples were drawn from the same distribution.

#### 2.2.3. SMOTE Data Augmentation to Overcome Class Imbalance

The main problem that the dataset considered is that it is highly imbalanced, i.e., only one among eight objects were targeted for detection. The ML classifier becomes biased toward the undesired majority class if the same data are provided for training. Hence, data must be balanced by using data augmentation techniques before giving it to train a classifier. There are many augmentation techniques existing in the literature [46]. Among the existing techniques, data sampling methods such as undersampling, oversampling, synthetic minority oversampling (SMOTE), etc., proved efficient and easy to implement. Undersampling removes data from the majority class to balance the majority and minority classes. This may lead to the loss of useful information. Oversampling repeats the minority class data to a level where both minority and majority classes balance each other. This may lead to the overfitting of a classifier. SMOTE [47] uses the K-nearest neighbor (KNN) algorithm to maintain data balance. This approach reduces the overfitting problem brought on by random oversampling. It focuses on the feature space and generates new instances by interpolating between positively correlated examples that are spatially close to one another. Hence, SMOTE is chosen to augment the data used in this research. This also overcomes the dataset class imbalance challenge in ML analysis that was performed in this work. After data augmentation using SMOTE, the data matrix size changes to 54,880 × 30. Now the dataset contains equal proportions of target and non-target data.

#### 2.2.4. Classification

ML classifiers are one of the best choices for performing classification tasks in many real-world applications. They are found to give superior performance when compared to other traditional classifiers.

In the present work, k-fold cross-validation was used. In this technique, all the available data were randomly divided into k sub-groups of equal size, out of which all the (k − 1) sub-groups were used for training, and the leftover group was used for testing. The final classification accuracy was the mean accuracy obtained from all the k-values during the cross-validation process. Here, k is taken to be 10. The main advantage of this cross-validation process is that it avoids the overfitting of the model. Three types of ML classifiers belonging to three different categories, namely the ensemble bagged tree (EBT), support vector machine with a fine Gaussian kernel (SVM (FG)), and artificial neural network (ANN), were used for training on each mode individually, and the performance parameters such as accuracy, F1-score, the area under the curve (AUC), sensitivity, specificity, and negative predictive value were calculated to access the performance of a classifier after testing.

#### 2.2.5. Machine Learning Classifiers

There are many ML algorithms for EEG signal classification. However, the three most popular ML classifiers, which are representative of three different classifier categories, namely, ensembles, vector machines, and neural networks, were considered for classification. The main reason behind selecting SVM, EBT, and ANN classifiers is that they were found to give very good performance in many of the recent works reported in the literature for classifying non-linear and non-stationary EEG signal data in BCI applications. For instance, in a recent work, [40] for BCI-based silent communication, SVM and EBT classifiers gave good classification accuracy compared to other contemporary machine learning algorithms such as K-nearest neighbors and decision trees. In another recent work [13] for BCI-based robot control, lightweight ANN provided a better performance compared to recurring neural networks. Moreover, SVM (FG), EBT, and ANN classifiers are relatively fast for BCI applications and are also very good at classifying non-linear P300-EEG data. All the above-mentioned reasons encouraged us to select SVM (FG), EBT, and ANN classifiers for our application. After extensive experimentation, the classifiers that were selected were also found to perform better compared to other classifiers in their respective categories. The classifiers chosen for the present work are clearly described below.

##### Ensemble Bagged Tree (EBT) Classifier

**Figure 9:**
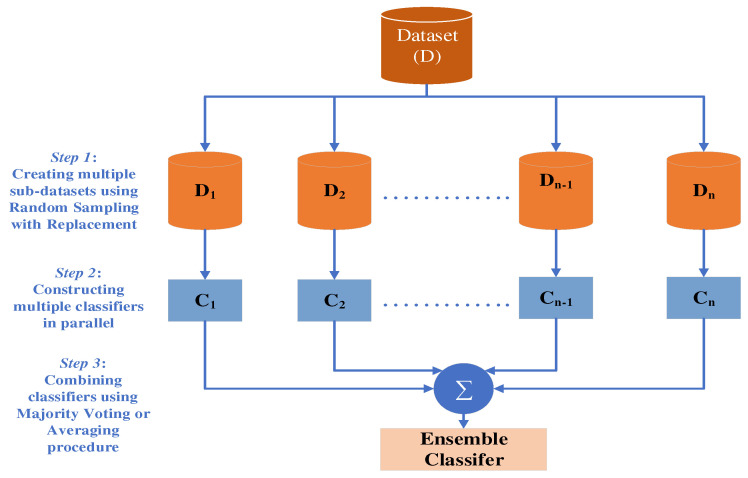
EBT Classifier.

The EBT classifier is an ensemble bagging of decision tree classifiers. A decision tree (DT) [48] is a supervised non-parametric learning method that is used for classification. Bagging, or bootstrap aggregating, proposed by Breiman [49], is a technique for creating multiple versions of a predictor and combining them to produce an aggregated predictor. Bootstrap resampling is used to create subsets from the training set of data in a bagged DT classifier, and each decision tree is built using a subset of the training data. The number of trees that are formed is determined by the number of bootstraps. Then, a majority voting procedure is used for the DT outputs trained on various subsets. Ensemble methods aim to improve the predictive performance of a given statistical learning or model fitting technique. The fundamental idea behind ensemble approaches is to create a linear combination of many model-fitting techniques [50]. Figure 9 illustrates the concept of ensemble bagging. The ensemble bagging, as shown in Figure 9, consists of three main steps. They are:

1. The training dataset D is divided into multiple sub-datasets $D_{1},D_{2}$…$D_{n}$ using random sampling with replacement.
2. Build multiple decision tree classifiers $C_{1},C_{2}$…$C_{n}$ by training sub-datasets $D_{1},D_{2}$…$D_{n}$ respectively.
3. Combine the resultant classifiers using the majority voting or averaging procedure to arrive at an ensemble classifier.

The equation below illustrates the mathematical concept of the ensemble method for classification

(7) $${\hat{C}}_{ens}\left(\cdot \right)=\sum_{j=1}^{n}w_{j}C_{j}\left(\cdot \right)$$

where $C_{j}\left(\cdot \right)$ represents the classifier function obtained after being trained on the sub-datasets, $w_{j}$ is the average weight, and ${\hat{C}}_{ens}\left(\cdot \right)$ is the ensemble-based function estimator.

##### Support Vector Machine with Fine Gaussian Kernel Classifier

**Figure 10:**
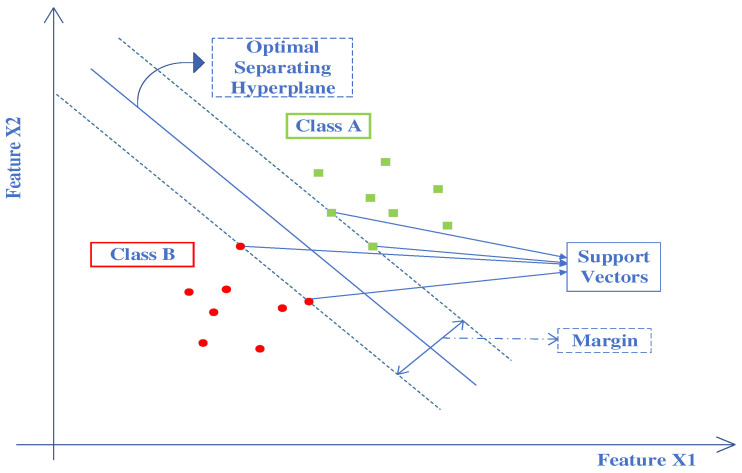
SVM classifier with hyperplane, support vectors and margin for two different classes.

SVM [51] is a renowned supervised ML algorithm. It is used for binary as well as multi-class classification. SVM uses the Lagrangian dual problem for optimization. It results in an increase in speed and a reduction in training time compared to other algorithms. Hence, SVM is highly effective not only for high-dimensional problems with fewer data but also for large datasets. It performs binary classification by arriving at an optimal hyperplane that separates observations belonging to one class from the other. For a linearly separable dataset, the support vectors with hyperplane are shown in Figure 10.

The Kernel function in SVM is used to transform data from a lower dimension to a higher dimension by which non-linear separation can be performed in that feature space. These Kernels are generally of three types, namely Gaussian, polynomial, and sigmoid. The Gaussian kernel function maps the input data to Hilbert space. It allows the separation of non-linearly separable data. The Gaussian kernel function is given by:

(8) $$K_{Gauss}\left(a,b\right)=\exp\left(-\frac{{∥a-b∥}_{2}^{2}}{2\rho^{2}}\right)$$

where *a* and *b* in (8) are input vectors. The numerator part of the exponential function in (8) is the Euclidean norm obtained with the input vectors, and ρ is a real constant in the denominator. The Gaussian kernel function decays equally across all directions surrounding the support vector. However, it decays exponentially in the input feature space and leads to the kernel function’s hyperspherical contours.

##### Artificial Neural Network Classifier

**Figure 11:**
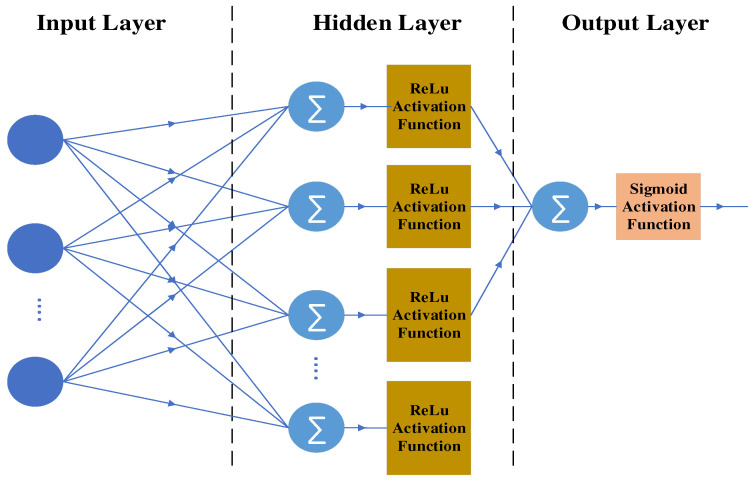
Artificial neural network architecture.

ANN is a lightweight, simple, fully connected feedforward ANN [52] architecture. The architecture used in the present work is shown in Figure 11. It consists of an input layer that accepts the input of 30 EEG feature samples. The outputs of the inputs are fed forward to the fully connected hidden layer of 100 neurons with a rectified linear activation unit (ReLu) activation function [53]. The number of hidden layers is chosen more than the number of inputs; hence, it can be categorized as a wide ANN. The ReLu activation function is used in the hidden layer as it eliminates the vanishing gradient problem and allows the network to perform better and faster. The outputs of the hidden layer are fed to the output layer. The output layer consists of a neuron with a sigmoid activation function [53] that is used for binary classification. The Sigmoid activation function outputs the probability of the input as a target or a non-target signal.

The most probable optimal hyper-parameters for all the above ML-based classifiers considered in this work is given in the Table 1.

## 3. Results

The problem at hand is a binary classification problem of identifying the target’s P300 signal and the non-target non-P300 signal. The primary tasks of the present work are to identify the optimal ML classifier to perform the classification task and the VMD mode, which shows optimal performance. Hence, the data are given for the training and testing of three types of ML classifiers using k-fold cross-validation (k = 10). The results obtained after extensive experimentation are explained in the following subsections.

### 3.1. Comparison of Classifier Performance over Different Modes

**Figure 12:**
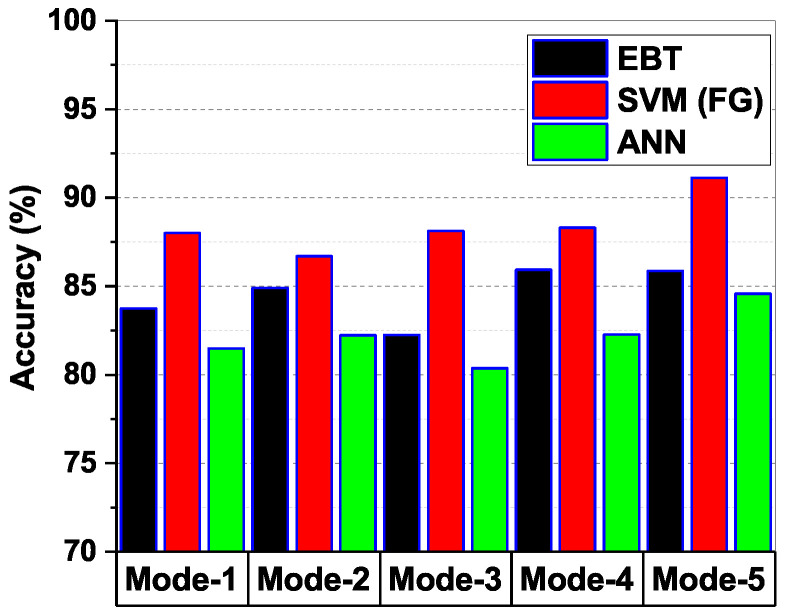
Comparison of modewise average accuracy for ML classifiers.

Figure 12 shows the average accuracy obtained from the testing data of each subject using the three classifiers EBT, SVM (FG), and ANN on all the five modes of VMD. From Figure 12, it can be seen that the SVM (FG) classifier performed better than other classifiers. It can also be inferred that among all the modes, the accuracy of Mode-5 was better compared to other modes using SVM (FG) classifier. The average classification accuracy of the SVM (FG) classifier on Mode-5 was 91.12 %. Hence, it can be inferred that the VMD-SVM combination on Mode-5 is optimal. According to [54], P300 is commonly found in low-frequency brain wave signals. It can be seen from Figure 8 that Mode-5 is a low-frequency signal compared to other modes. These findings thus strongly support the findings in [54] that P300 signals occur in lower frequency bands on the EEG signal.

### 3.2. Comparison of Subject Wise Average Accuracy for Different Classifiers on Mode-5 Signal

**Figure 13:**
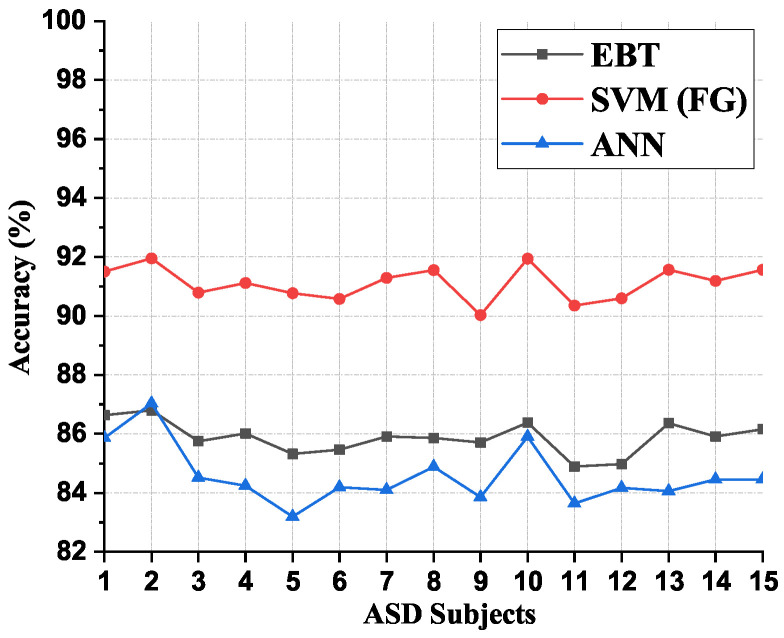
Comparison of subject wise average accuracy for EBT, SVM (FG), and ANN classifiers on the Mode-5 signal.

Figure 13 shows a comparison of the subject-wise average accuracy for the classifiers considered in the present work. It is evident from Figure 13 that the SVM (FG) classifier gave consistently good classification accuracies irrespective of the subjects considered when compared to EBT and ANN classifiers. This also illustrates the stability of the performance of the SVM (FG) classifier.

### 3.3. Mode-Wise Comparison of Average of Performance Parameters (%) for the Classifiers on Each Mode

**Figure 14:**
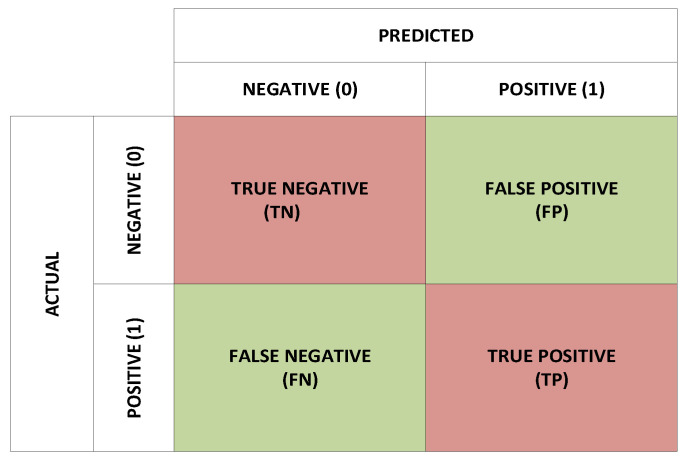
Confusion matrix.

The average values of vital performance parameters other than accuracy, such as sensitivity, specificity, precision, F1-score, AUC, and the negative predictive value (NPV), were calculated to assess the best-performing VMD-SVM method further. The general structure of the confusion matrix is shown in Figure 14. The performance evaluation parameters are computed from the confusion matrix using the following formulae:

(9) $$Sensitivity=\frac{TP}{TP+FN}$$

(10) $$Specificity=\frac{TN}{TN+FP}$$

(11) $$Precision=\frac{TP}{TP+FP}$$

(12) $$F1\;Score=\frac{TP}{TP+\frac{1}{2}\left(FP+FN\right)}$$

(13) $$Negative\;predictive\;value=\frac{TN}{TN+FN}$$

**Figure 15:**
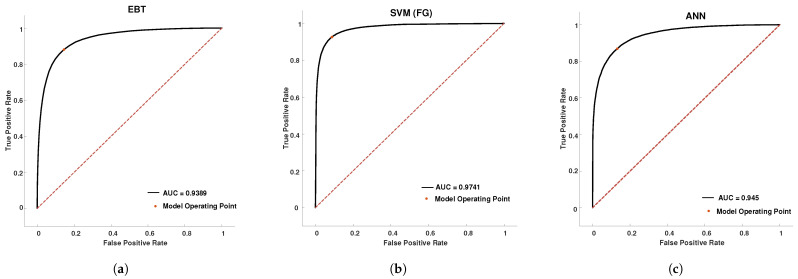
ROC curves for cross-validated testing data of subject-2 Mode-5 signal for (**a**) the EBT classifier, (**b**) SVM (FG) classifier, and (**c**) ANN classifier.

A mode-wise comparison for all the classifiers considered is presented in Table 2. As can be observed from Table 2, the performance parameters for SVM (FG) on the Mode-5 signal is superior compared to EBT and ANN classifiers. In the case of SVM (FG) on Mode-5, the average F1 score was 91.18%, the average AUC was 96.6%, the average sensitivity was 91.79% the average specificity was 90.41%, the average precision was 90.54%, and the average NPV was 91.70%. The sample cross-validated testing ROC curves for the classifiers on the Mode-5 signal of subject-2 are shown in Figure 15 respectively. The red dashed line in Figure 15 is the line of reference.

After observing these results, it can be concluded that the SVM (FG) classifier on the VMD Mode-5 signal outperformed other classifiers considered in this work.

## 4. Discussion

In this work, the main aim was to efficiently classify P300 signals from non-P300 signals in ASD subjects to help design an efficient BCI system for neuro-rehabilitation training, which is a vital personalized therapeutic approach. An adaptive signal decomposition-based technique was explored to achieve this task by looking at the peculiarity of the P300 signal in ASD subjects. The VMD-based decomposition decomposes the P300 signal into five constituent modes. Then, each one of the five mode’s features is extracted and given a classification for three ML classifiers, EBT, SVM (FG), and ANN. After experimentation, it was found that the low-frequency Mode-5 signal gave a good performance classification compared to other modes. After that, the performance of the ML algorithms on specific Mode-5 data were assessed, and it was found that SVM (FG) outperformed EBT and ANN classifiers. Hence, our findings show that the VMD Mode-5 signal combined with SVM (FG) performed well compared to other classifiers.

Table 3 shows the comparison of the accuracy of the proposed technique compared with the other cutting-edge techniques. The accuracy of the proposed VMD-SVM method was 91.12% which is better than the other state-of-the-art methods with similar hardware and comparable to the deep learning (DL) methods using superior hardware. The execution time of the proposed VMD-SVM model is around 0.37 ms on average over a personal computer (PC) with 16 GB RAM and Intel(R) Core(TM) i5-8500 CPU. This execution time is much less than other state-of-the-art methods using comparable hardware specifications. This makes the proposed method an attractive candidate for real-time automatic BCI implementation. For efficient BCI application, the classifier must also be trained quickly as far as possible. The computation complexity for training the proposed VMD-SVM method is lower than that of other machine learning methods.

The advantages and drawbacks of the proposed method compared to other studies are summarized in Table 4. These advantages make the proposed method more interesting for online BCI applications. Therefore, the proposed VMD-SVM method has good potential in BCI-based neuro-rehabilitation training to assist people with ASD.

## 5. Conclusions

VR-based P300 BCI has gained importance for neuro-rehabilitation training to assist ASD individuals. Developing an automatic and real-time optimal model for P300 signal identification is a crucial step in designing a practical BCI model for ASD. The current work was the first pilot investigation to classify P300 signals from non-P300 signals in ASD participants using the VMD adaptive signal decomposition algorithm and ML classifiers. As the adaptive selection of the basis function considerably minimizes the decomposition error and preserves information in the original signal, the use of adaptive signal decomposition for building BCI is very encouraging. For the proposed VMD-SVM method, accuracy is on par with other cutting-edge methods, and the training and classification time is less for the same classification task. Thus, the proposed VMD-SVM method proved efficient and can be employed to build a real-time BCI to assist ASD subjects. The actual application’s VMD-SVM method may also be used in BCI applications which can be used to generate imagined words from EEG signals, including wheelchair control for paralyzed individuals, and to assist subjects with Amyotrophic lateral sclerosis who suffer from a lack of muscle movement.

### Future Scope

The necessity to know in advance how many modes and how much data are to be binned is a classic shortcoming of many adaptive signal decomposition methods such as VMD [39] used in this work. In this work, the EEG signal is split into five modes only because the EEG signal, in general, has five frequency bands associated with it. Extracting the exact number of VMD modes that are required to achieve maximum performance is also an important area to explore. Decomposition into a smaller number of modes would result in mode mixing, whereas decomposition into more number of modes would result in mode redundancy. Hence, the utilization of optimization algorithms for mode optimization needs to be explored. The automatic identification of the number of modes needed in VMD also facilitates the design of a fully automatic BCI system in future research. The availability of more open-source datasets for ASD would have facilitated us in further testing the efficiency of the proposed model. The optimal number of feature selections may also be explored in future studies to reduce the data needed for training and testing. DL classifiers and their optimization for ASD also need to be explored.

## Figures and Tables

**Table 1:** Hyper-parameters of ML classifiers.

ML Classifier	Hyper-Parameters	Description
EBT	Preset	Bagged Trees
	Ensemble method	Bag
	Learner type	Decision Tree
	Maximum number of splits	54,879
	Number of learners	30
	Number of predictors to sample	Select All
SVM (FG)	Preset	Gaussian SVM
	Kernel function	Gaussian
	Kernel scale	1.4
	Box constraint level	1
	Multiclass method	One-vs-one
	Standardize data	Yes
ANN	Preset	Wide neural network
	Number of fully connected layers	1
	First layer size	100
	Activation	Rectified linear unit(ReLU)
	Iteration limit	1000
	Regularization strength	0
	Standardize data	Yes

**Table 2:** Modewise comparison of the average performance supporting parameters for ML classifiers.

Mode No.	Classifier	Sensitivity (%)	Specificity (%)	Precision (%)	F1-Score (%)	AUC (%)	NPV (%)
1	EBT	85.41	82.09	82.67	84.02	91.46	84.92
	SVM (FG)	89.78	86.22	86.70	88.21	94.60	89.40
	ANN	80.24	82.72	82.28	81.25	90.00	80.73
2	EBT	86.38	83.41	83.90	85.12	92.53	85.96
	SVM (FG)	89.65	83.73	84.65	87.08	93.53	88.95
	ANN	80.78	83.68	83.19	81.96	90.80	81.34
3	EBT	86.90	83.60	84.13	85.49	92.73	86.44
	SVM (FG)	91.09	85.13	85.98	88.47	94.66	90.48
	ANN	80.15	80.59	80.52	80.33	88.93	80.24
4	EBT	86.51	85.35	85.52	86.01	93.20	86.35
	SVM (FG)	90.23	86.38	86.89	88.53	94.93	89.84
	ANN	81.47	83.07	82.81	82.13	90.60	81.69
5	EBT	87.27	84.47	84.90	86.12	93.33	86.90
	SVM (FG)	91.79	90.41	90.54	91.18	96.60	91.70
	ANN	83.97	85.16	84.97	84.47	92.53	84.16

**Table 3:** Comparison of accuracies (%) for the proposed VMD-SVM model with other state-of-art methods.

Author	Method	Accuracy (%)
Borra et al. [35]	CNN Based on EEG-NET	92.30
Santamaría-Vázquez et al. [34]	CNN-BLSTM	84.30
Lucia de Arancibia et al. [33]	LDA with time and CWT features	82.00
Bittencourt-Villalpando et al. [32]	Linear LDA with pseudo-random averaging	81.20
Miladinovic et al. [31]	Logistic regression based on variational Bayesian inference	80.30
Bipra Chatterjee et al. [30]	Temporal features with Bayes LDA	76.30
Adama et al. [29]	Time domain features and Pearson correlation coefficient	70.00
Proposed	VMD-SVM	91.12

**Table 4:** Advantages and drawbacks of the proposed method compared to other studies.

Advantages	· Accuracy of the proposed VMD-SVM method is 91.12%. It outperforms other cutting-edge techniques using similar hardware. · Accuracy is comparable to the deep learning (DL) methods using superior hardware. · Execution time of around 0.37 ms on average over a PC with 16 GB RAM and Intel(R) Core(TM) i5-8500 CPU. · Training time of around 50 min on average using the same hardware mentioned above. · Execution and training times are much smaller than other state-of-the-art methods using comparable hardware specifications. · Computation complexity for training the proposed VMD-SVM method is lower than that of other machine learning methods.
Drawbacks	· Automatic feature extraction from the raw EEG data may be performed the same as other state-of-the-art DL architectures. · Automatic identification of the optimal number of VMD modes can be performed.

## Data Availability

The BCIAUT-P300 benchmark dataset used in this research work is publicly available and can be found at https://www.kaggle.com/datasets/disbeat/bciaut-p300 (accessed on 18 January 2023).
